# Understanding Long-Term Variations in an Elephant Piosphere Effect to Manage Impacts

**DOI:** 10.1371/journal.pone.0045334

**Published:** 2012-09-17

**Authors:** Marietjie Landman, David S. Schoeman, Anthony J. Hall-Martin, Graham I. H. Kerley

**Affiliations:** 1 Centre for African Conservation Ecology, Department of Zoology, Nelson Mandela Metropolitan University, Port Elizabeth, South Africa; 2 Faculty of Science, Health, Education and Engineering, University of the Sunshine Coast, Maroochydore, DC, Queensland, Australia; 3 Hall-Martin Consulting CC, Somerset West, South Africa; Australian Wildlife Conservancy, Australia

## Abstract

Surface water availability is a key driver of elephant impacts on biological diversity. Thus, understanding the spatio-temporal variations of these impacts in relation to water is critical to their management. However, elephant piosphere effects (i.e. the radial pattern of attenuating impact) are poorly described, with few long-term quantitative studies. Our understanding is further confounded by the complexity of systems with elephant (i.e. fenced, multiple water points, seasonal water availability, varying population densities) that likely limit the use of conceptual models to predict these impacts. Using 31 years of data on shrub structure in the succulent thickets of the Addo Elephant National Park, South Africa, we tested elephant effects at a single water point. Shrub structure showed a clear sigmoid response with distance from water, declining at both the upper and lower limits of sampling. Adjacent to water, this decline caused a roughly 300-m radial expansion of the grass-dominated habitats that replace shrub communities. Despite the clear relationship between shrub structure and ecological functioning in thicket, the extent of elephant effects varied between these features with distance from water. Moreover, these patterns co-varied with other confounding variables (e.g. the location of neighboring water points), which limits our ability to predict such effects in the absence of long-term data. We predict that elephant have the ability to cause severe transformation in succulent thicket habitats with abundant water supply and elevated elephant numbers. However, these piosphere effects are complex, suggesting that a more integrated understanding of elephant impacts on ecological heterogeneity may be required before water availability is used as a tool to manage impacts. We caution against the establishment of water points in novel succulent thicket habitats, and advocate a significant reduction in water provisioning at our study site, albeit with greater impacts at each water point.

## Introduction

Herbivores influence vegetation dynamics at a range of spatial and temporal scales, with the intensity and heterogeneity of these effects determined by a spatio-temporal hierarchy of foraging decisions. At a landscape scale, foraging decisions made at finer-scales are constrained by both biotic and abiotic factors, including proximity to water, topography and the availability and quality of food [1]. For elephant (*Loxodonta africana*), surface water availability is a key limiting resource that influences population dynamics, movement and range-use patterns [2]–[4], and hence impacts on biological diversity. Consequently, much of the debate around managing the impacts of southern Africa’s elephant population has focused on the management of surface water, particularly when supplemented (e.g. through boreholes) away from natural permanent water [5], [6]. Where elephant movements are modified by the provision of artificial water points [3], [4], effects on vegetation dynamics become more widespread, and intensify in areas that previously functioned as seasonal refuges for plant regeneration [7]. Conservation areas with abundant water supply and elevated elephant numbers are therefore vulnerable to degradation as the utilization gradients that develop around water coalesce and vegetation structure is homogenized across the landscape [7]–[9]. These changes have severe implications for other herbivores (e.g. [10], [11]) and presumably other elements of biodiversity, with consequences for ecosystem processes and resilience [7]. Thus, developing a predictive understanding of the spatial and temporal variations of elephant impacts in relation to water is key to managing these impacts. This comes at a time when conservation managers use water availability as a tool to manipulate elephant distributions in an attempt to maintain landscape heterogeneity [5], [9], [12].

The (foraging and trampling) impacts of herbivores on vegetation dynamics and soil resources in relation to water, creating a piosphere effect (i.e. a radial pattern of attenuating impact), are well documented, particularly for rangelands (reviewed in [13]). Descriptive models of these spatial patterns are expected to show sigmoid responses, which are intuitively attractive as tools to estimate the extent of the piosphere effect (on the basis of the distance from water at which the asymptote of the curve is reached), and thus to determine the location of water points (allowing for areas of imperceptible impacts) across the landscape [14], [15]. For herbaceous communities, these distances vary with rainfall, herbivore numbers and the proximity of neighboring watering points (e.g. [16], [17]), but this has not been tested for woody communities that may be less dynamic; neither is it clear how the extent of the piosphere effect contrasts between communities or features within a community (e.g. biomass vs. abundance) that may differ in their sensitivity to impacts. Our understanding of these variations is confounded by a lack of long-term quantitative studies on spatio-temporal variations in piospheres (e.g. [18]), while conceptual models were developed for open (i.e. non-fenced) rangelands [14]. In these systems, sigmoid models are expected to show increasing displacement of asymptotes away from water (allied with declining curve steepness) as the piosphere pattern expands with continuous utilization [8], [14]. However, many wildlife systems, and particularly those with elephant, are more complex (i.e. fenced, multiple water points, seasonal water availability, varying population densities) such that the predicted spatio-temporal variations may not always hold [19], thus questioning the reliability of these models as management tools.

Despite the documented changes in vegetation structure and dynamics caused by elephant (reviewed in [20]) and the fact that these impacts intensify near water (e.g. [2], [4], [21]), elephant piosphere effects are poorly described or simply inferred. Moreover, these descriptions are largely restricted to savanna habitats and most focus on herbaceous communities that appear to be resilient to impacts (e.g. [16], [17], [22]); these communities also respond strongly to other environmental drivers (e.g. drought, rainfall variability), such that our understanding of the impacts may be confounded [20], [23]. Thus, despite concerns that vegetation structure may be homogenized across landscapes with consequences for ecosystem processes, few studies [19], [21], [24]–[26] have considered the components of the vegetation (i.e. woody shrubs and trees) that are likely to show long-term responses and are vulnerable to elephant effects. This highlights the need to demonstrate elephant piosphere effects across a range of habitats, focusing on the woody components.

Using 31 years of data on shrub structure in the succulent thickets of the Addo Elephant National Park (AENP), South Africa, we test spatial and temporal variations in elephant impacts at a single water point. We predict that shrub structure increases rapidly to an asymptote with distance from water, a classic piosphere effect, but that the extent of the effect varies depending on the sensitivity of the structural feature to elephant impacts [14]. With time and increasing elephant numbers, we expect the piosphere effect to expand (characterized by an increasing displacement of asymptotes away from water and declining curve steepness – [8], [14]) as the shrub community is gradually replaced with a community of grasses. Because shrub structure is important for ecological functioning (*sensu* [27]) in succulent thicket [28], [29], this change would be expected to cause a loss in functionality, particularly in areas adjacent to water. We hypothesize that this pattern can be interpreted in terms of a state-and-transition model and show that elephant have the ability to expand the grassland-state across the landscape, causing severe transformation. Finally, we argue that in fenced areas (created through physical or figurative barriers – [30]) with abundant water supply, elephant piosphere effects are complex, which in the absence of long-term data and careful, scientific design of monitoring programmes, limits our ability to predict and manage these impacts.

### Study Area

**Figure 1:**
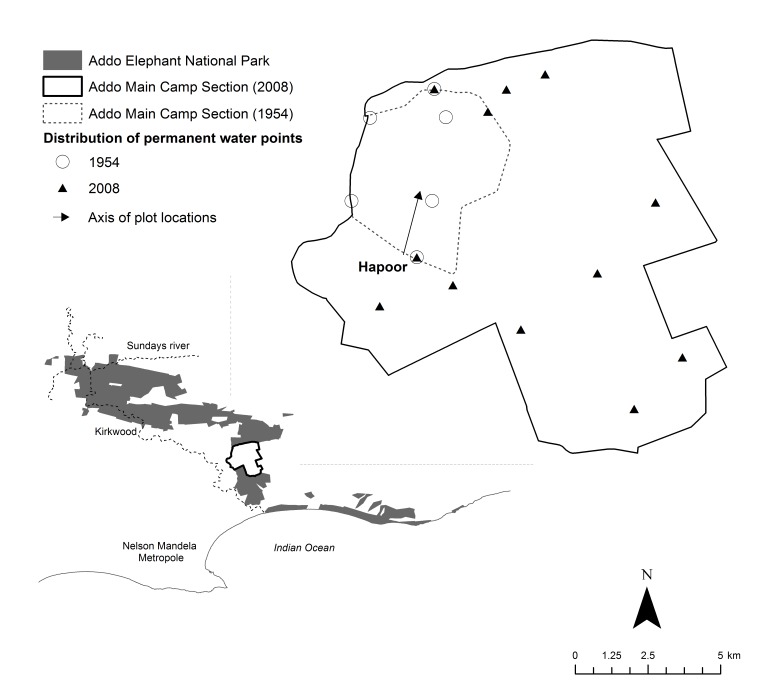
Location of water points in the Addo Main Camp section (study area), Addo Elephant National Park. Experimental plots were placed at increasing distances from Hapoor water point in the area originally fenced in 1954. The incremental expansion of AMC caused a substantial increase in the number of permanent artificial water points (from 6 in 1954 to a total of 12 in 2008); only two of these (shown by overlapping symbols) maintained water availability for elephant since the initial fencing.

AENP (33°31’S, 25°45’E) is located in the Eastern Cape Province, South Africa (Fig. 1). The park comprises0020pseveral fenced sections with the majority of the elephant population confined to the Addo Main Camp section (AMC; 120 km^2^ at the time of the study). AMC was originally fenced in 1954 (23.3 km^2^) to enclose the elephant of the region and incrementally expanded to accommodate growing numbers (from 22 individuals in 1954 to 384 in 2008; [31]). The area also supports a diverse ungulate community (12 spp.), but elephant contribute the bulk (*∼*80%) of herbivore biomass [32].

The region is semi-arid with 260–530 mm rainfall annually, spread throughout the year, with small peaks in spring and autumn. Because no natural permanent surface water is available in AMC, a large number of artificial water points (pumped point sources) were established (from 6 in 1954 to a total of 12 in 2008; Fig. 1). The terrain comprises a series of low, undulating hills (60–350 m in height) in the Sundays River valley, where nutrient-rich soils give rise to succulent thicket habitats [33]. Herbivory is considered the key driver of thicket structure, with rainfall and fire playing relatively minor roles [34]. These thickets are typically evergreen, 2–4 m high, dense and characterized by a high diversity of growth forms [33]. The tree succulent *Portulacaria afra* is locally dominant and occurs in a matrix of spinescent shrubs (e.g. *Azima tetracantha*, *Capparis sepiaria, Carissa bispinosa*, *Gymnosporia* spp., *Searsia* spp.) and low trees (e.g. *Euclea undulata*, *Schotia afra, Sideroxylon inerme*). Although grasses are usually sparse [35], couch grass *Cynodon dactylon* may be seasonally abundant where intensive utilization by elephant has removed the canopy shrubs.

## Methods

### Ethics Statement

N/A

### Vegetation Structure

We measured the composition and structure (defined in terms of shrub volumes and densities) of the thicket shrub community along seven experimental plots located at increasing distances (100, 200, 300, 500, 1000, 1500 and 3000 m) from Hapoor water point in the area of AMC originally fenced in 1954 (Fig. 1). Hapoor represents one of only two water points that have maintained water availability for elephant since the initial fencing. Plots were permanently marked in 1977, when they were first surveyed, with further monitoring in 1981, 1989 and 2008 (providing temporal coverage of 31 years). Thus, the sampled plots experienced 23–54 years of elephant use over the experimental period, at a time when densities fluctuated between 1.0 and 4.1 elephant.km^−2^.Since succulent thicket is an aseasonal habitat with an evergreen shrub community [35], we did not consider any seasonal variations in elephant effects.

Plots were 5 m wide, while plot length (17–45 m) scaled inversely with the abundance of the dominant shrub taxa. We estimated the volume (m^3^.m^−2^) of all canopy shrubs (24 spp.: 5 succulents, 19 woody shrubs) encountered by measuring the maximum height and canopy diameters of individual plants. Because most shrubs are multi-stemmed re-sprouters, stems within 50 cm of each other at ground-level were considered to be of the same individual. Individuals were measured if at least half the rooted area occurred within the plot. We calculated shrub density as the number of individuals per unit area.

### Ecological Functioning

According to the landscape functionality framework of Ludwig, Tongway, Freudenberger, Noble and Hodgkinson [27], landscapes that capture resources (e.g. organic matter, soil material) are more functional than those where such resources are lost. In succulent thicket, resources are captured and retained beneath patches of canopy shrubs (forming raised organic-rich mounds - e.g. [28]), such that the loss of these patches causes a smoothing of the soil surface as resources, and hence functionality [28], [29], is lost. Using these predictions, we estimated ecological functioning at increasing distances from water by measuring areas of run-on (i.e. convex soil surface) and run-off (i.e. concave soil surface; adapted from [27]) along three 50 m line-transects located at each marked experimental plot. Results are presented as the ratio between areas of run-on and run-off per plot.

To identify the likely mechanism of the predicted change in functionality, we hypothesized that this process will be associated with a change in the structure (or integrity) of the organic-rich mounds that occur beneath patches of canopy shrubs. Hence, we considered intact mounds to be those for which patch area was equal to, or exceeded mound area and thus where resources were conserved beneath patches. The reverse was true for exposed mounds; these occurred more frequently near water, reflecting areas vulnerable to erosion. Thus, at each marked experimental plot we measured the canopy and mound diameters of ten randomly selected shrub patches and estimated patch and mound area (m^2^), respectively. Ratios of patch and mound area were correlated with ratios of run-on and run-off per plot.

### Intensity of Use

Our approach assumed that elephant were the key drivers of vegetation structure and ecological functioning in AMC, and ignored the effects of other herbivores. Although this reflected our observations of the scale and magnitude of impacts on the shrub community (determined by the versatile and destructive foraging of elephant – e.g. [23], [31], [32]), we validated this approach by estimating the relative intensity of use by herbivores at increasing distances from Hapoor water point. During the final survey we conducted standing-crop dung counts [36] for all herbivores encountered at each experimental plot (area standardized to 250 m^2^). Because counts were generally poorly distributed across plots for individual species, these were combined across species to estimate herbivore densities and for comparison with estimates of elephant density. Dung counts have been shown to provide reliable estimates of relative use between elephant and other herbivores [37], [38].

### Data Analysis

Data for the 1977, 1981 and 1989 surveys were available from Barratt and Hall-Martin [39].

Non-metric Multidimensional Scaling (n-MDS) ordinations, based on Bray-Curtis resemblance matrices of shrub density data [40], [41], were used to visualize differences in community composition over the experimental period. Data were square-root transformed to reduce the influence of extremely dominant species, and the fit of the ordination assessed with a *Stress* value. Each point on a biplot represents the data from a single experimental plot. Ordination analyses were performed with *Primer* Version 6 [41]. Plant nomenclature follows SANBI [42].

Using our conceptual understanding of the shape of the piosphere pattern and published examples (e.g. [14], [15]) we followed Crawley [43] in modeling trends in shrub volume and density using non-linear mixed-effects models (package nlme in R2.12.1; [44]) based on logistic growth curves [45], with Sample Period (four levels: 1977–2008) as a grouping variable. These curves comprised three fixed parameters and were of the form.
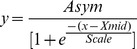
where *Asym* is the asymptote, *Xmid* is the curve inflection point and *Scale* is the magnitude of the dispersion of the function (i.e. the inverse of curve steepness ). Because we had no *a priori* information on the random effects variance-covariance structure of the models, we initially associated random effects for each Sample Period with all the fixed parameters (i.e. *Asym, Xmid* and *Scale*). Where there were indications of model over-parameterization [45], model selection proceeded by systematically removing non-significant random effects and comparing models using Akaike’s information criterion (*AIC*) and standard likelihood-ratio tests (*α* = 0.05). Superior models were indicated by a lower *AIC* value and significant tests [46]. Diagnostic plots of observed and fitted values and residuals were inspected for deviations from model assumptions. No formal method exists for post-hoc comparisons in the nlme package. Therefore, once a final model was selected for a set of variables, we assessed the importance of Sample Period by iteratively manipulating level codes for this variable to create groups, and determining the corresponding *AIC* for the modified model (with the same number of parameters as the original model). Modified models that decreased *AIC* by at least two (relative to the original model; [46]) were considered to improve the explanatory power.

Because shrub structure varied non-linearly with distance from water and we expected a clear relationship between ecological functioning and shrub structure, we modeled trends in functionality with a simple self-starting logistic growth curve.

## Results

### Intensity of Use

We recorded the dung of 10 mesoherbivore species at the experimental plots during 2008, but detected no relationship between dung densities for these species and distance from water (*R^2^* = 0.03; *F*_1,5_ = 0.14; *p* = 0.726). In contrast, dung densities for elephant, and thus intensity of utilization, declined exponentially (*R^2^* = 0.96; *F*_1,5_ = 116.70; *p*<0.001; Dung density = 0.07*e ^−0.01 * Distance to water^). This validated our approach and suggested that any piosphere effect observed was likely due to the effects of elephant.

### Vegetation Structure

**Figure 2:**
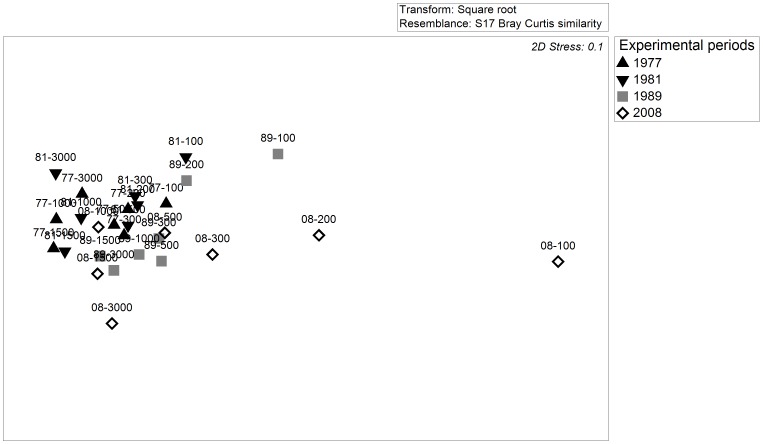
Non-metric Multidimensional Scaling ordination of the change in shrub composition over the experimental period (1977–2008). Sample codes refer Sample Period-Distance to water (m).

**Figure 3:**
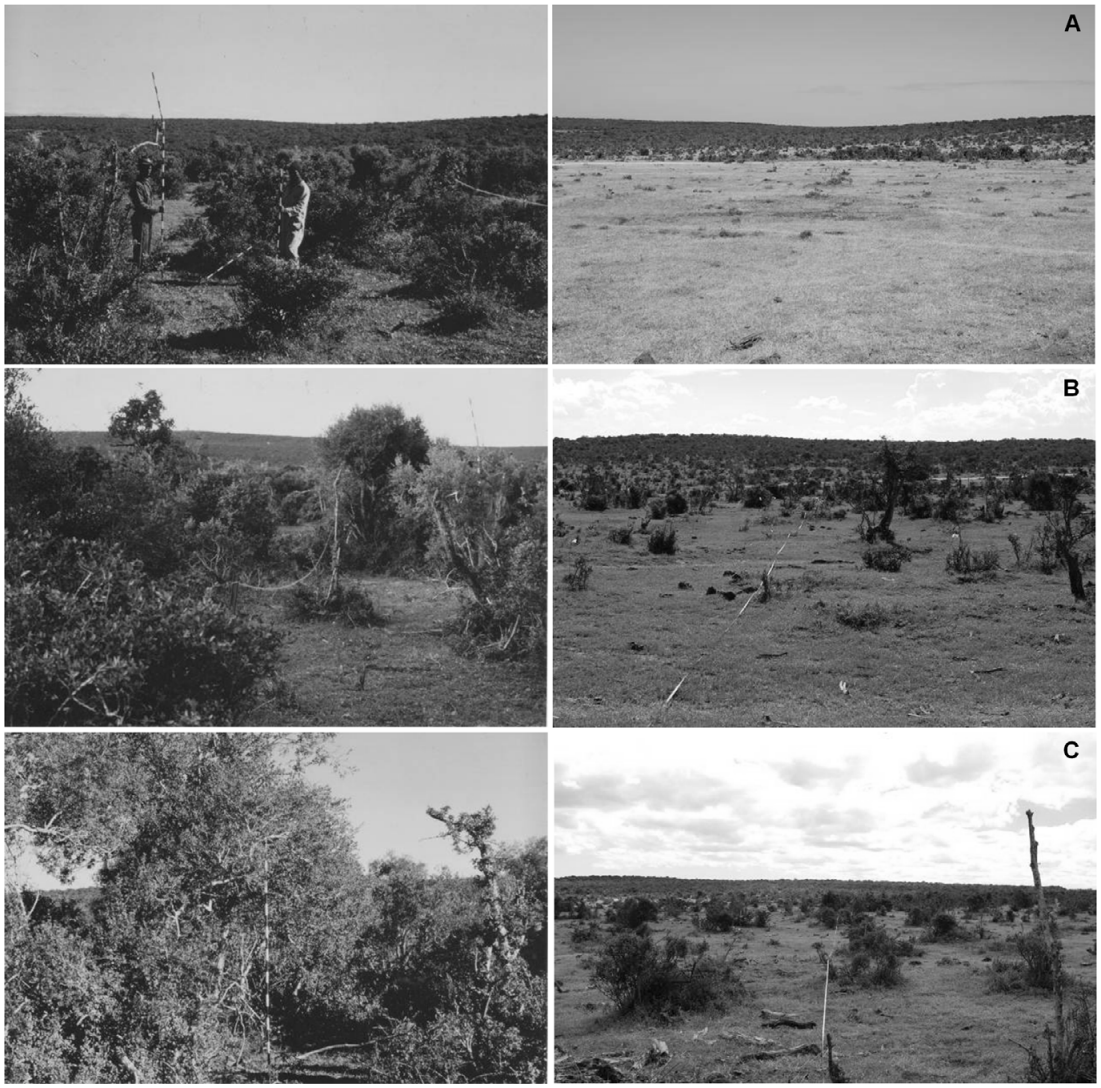
Contrasts in experimental plots located at 100 m (A), 200 m (B) and 300 m (C) from Hapoor water point between 1981 (left) and 2008 (right). Photo credits: M. Stalmans (1981), M. Landman (2008).

**Figure 4:**
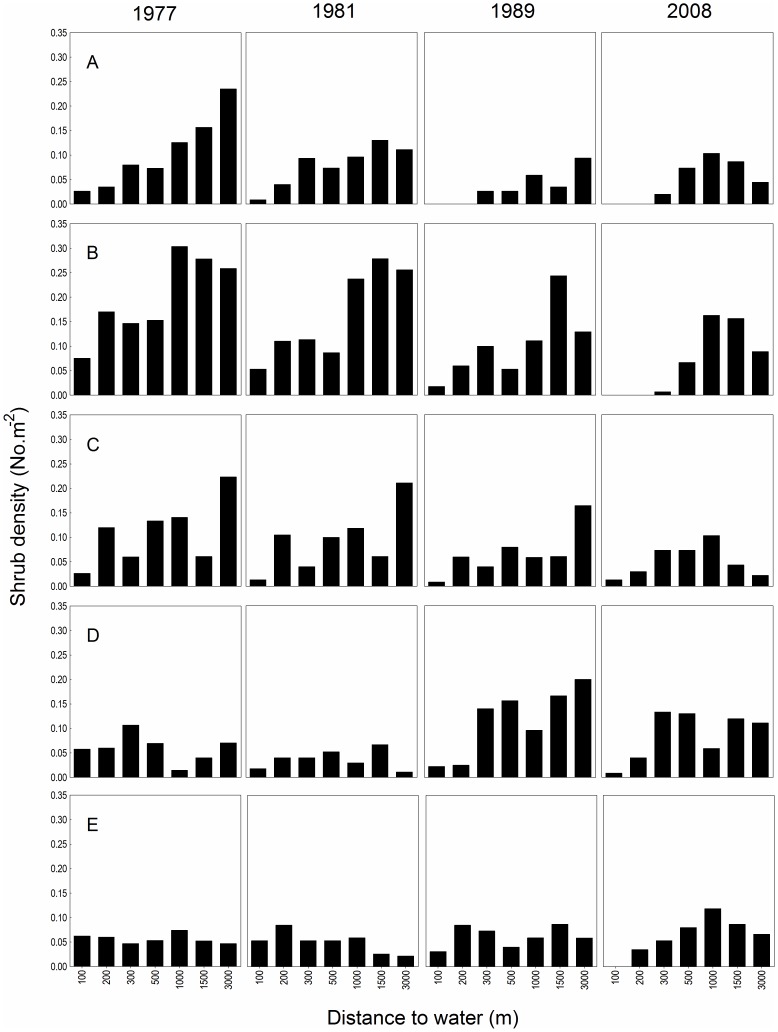
Trends in the density of the five dominant canopy species (A – *Portulacaria afra*, B – *Euclea undulata*, C – *Schotia afra*, D – *Azima tetracantha*, E – *Capparis sepiaria*) at increasing distances from water over the experimental period.

The n-MDS ordination showed a trend of increased dissimilarity in shrub community composition over the experimental period, largely determined by the effects of elephant near water (100–300 m; Fig. 2). These changes could be described at two levels. First, an assessment of the cover of canopy shrubs and grasses (along 50 m line-transects) at each marked experimental plot showed that intensive utilization by elephant caused the replacement of the shrub community with a community of grasses (Fig. 3). This meant that during the final survey, 92.3% of the landscape at 100 m from water comprised grasses (specifically *C. dactylon*). Grass cover declined exponentially with distance from water (*R^2^* = 0.84; *F*_1,5_ = 26.63; *p* = 0.004; % Grass = 1.02 * *e* ^−0.001 * Distance to water^). Second, within the shrub community, individual species responded differently to elephant effects (Fig. 4). For example, amongst the five canopy dominants for which we had sufficient data, *P. afra* appeared to be particularly vulnerable, showing a decline along the entire water gradient over the experimental period and disappearing from plots <300 m from water by 1989. Following the disappearance of more vulnerable species, shrub communities near water were dominated by *C. sepiaria* and *A. tetracantha*; the former appeared to resist removal, while the latter may have benefitted from being utilized (Fig. 4).

**Figure 5:**
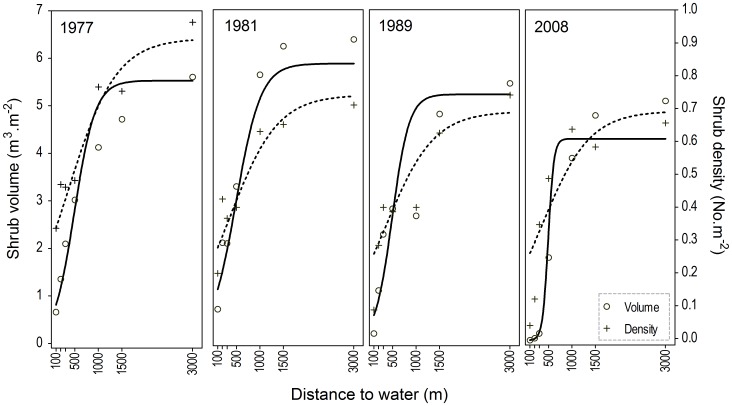
Best-fit mixed-effects logistic growth models of canopy shrub volume (solid lines; circles) and shrub density (dashed lines; crosses) as a function of distance from water.

Our mixed-effects models are based on only seven estimates of shrub volume or density at each of four years, which precluded the estimation of confidence intervals for individual parameter estimates. For this reason, although the results of our hypothesis tests are robust, care must be taken in over-interpreting the estimates of coefficients for individual years. Baring this in mind, our models showed a clear spatial pattern in shrub volume and density that is typical of piospheres (Fig. 5). For both response variables, model fit improved when model parameters were allowed to vary with Sample Period. Using this parameterization, *Asym* (i.e. the asymptote) and *Scale* (i.e. the inverse of curve steepness) varied significantly with time for shrub volume, while only *Asym* varied for density (Table 1). Contrary to our predictions, the displacement of asymptotes generally declined over the experimental period (volume: 59.6%; density: 6.0%) and were reached at distances between 2650 m (1977) and 1070 m (2008) from water for volume and between 4000 m (1977) and 3760 m (2008) for density (Fig. 5; Table 1). Further modeling by grouping of Sample Periods revealed differences between all asymptotes for shrub volume (i.e. the best model had separate parameter estimates for each Sample Period), with estimates for the 1981 survey being the highest (Table 1). *Asym* estimates for density, however, appeared to stabilize post-1981. Increased curve steepness with time post-1981 (72.8%; Table 1), allied with decreased asymptotes and constant inflection points, implied a radial expansion of the area adjacent to water with severely reduced shrub volumes (Figs. 3 and 5). Estimates from the mixed-effects models showed that during 1989, volumes at plots between 100–300 m from water had declined by 33.0–17.8% (Table 2). However, during 2008, these reductions reached 90% across the same plots such that grass cover in this area ranged between 92.3–75.6%.

**Table 1:** Best-fit mixed-effects logistic growth model selection results and parameter estimates for canopy shrub volume, shrub density and ecological functioning.

Best model parameters					Parameter estimates					
Fixed effects		Random effects	*K*	*Δ AIC*_1_	Sample period	Coefficient			*Δ AIC*_2_	
						*Asym*	*Scale*	*Xmid*		
**Shrub volume**										
	Asym + Xmid + Scale	Asym, Scale	8	−5.67	1977	5.53	222.02	489.75	15.69	
					1981	5.86	265.02	489.75	6.38	
					1989	5.22	181.61	489.75	15.69	
					2008	4.27	60.31	489.75	−1.97	
**Shrub density**										
	Asym + Xmid + Scale	Asym	6	−5.14	1977	0.92	511.30	361.95	−2.67	**
					1981	0.74	511.30	361.95	9.31	
					1989	0.69	511.30	361.95	9.26	
					2008	0.69	511.30	361.95	9.31	
**Run-on:Run-off area**										
	Asym + Xmid + Scale	Asym, Xmid, Scale			2008	4.43	470.00	838.33		
**Proportion of intact mounds**										
	Asym + Xmid + Scale	Asym, Xmid, Scale			2008	0.91	59.34	312.44		

Coefficients vary by Sample Period where they differ significantly from population coefficients, while non-significant coefficients are represented only by the population value. Coefficients were considered significantly different (*p*<0.05) from zero.

*Asym*, Asymptote; *Xmid*, Curve inflection point; *Scale*, Inverse of curve steepness.

*K,* Number of model parameters; *AIC,* Akaike information criterion; *ΔAIC*_1_, *AIC* difference between the full model with random effects for each Sample Period associated with all fixed parameters and the best model with a reduced random effects structure; *ΔAIC*_2_, *AIC* difference between a model with separate parameters for each Sample Period and a model with separate parameters for the selected period only;

** Sample Period different from all other periods combined.

**Table 2:** Percentage change in canopy shrub volume and shrub density over the experimental period as predicted by mixed-effects logistic growth models (see Fig. 5; Table 1).

Distance to water (m)	Percent change			
	1977∶1981	1977∶1989	1977∶2008	
**Shrub volume**				
100	34.5	−33.0	−99.2	
200	24.8	−25.4	−97.1	
300	16.6	−17.8	−89.3	
500	5.7	−5.2	−18.1	
1000	1.8	−2.1	−15.0	
1500	4.9	−5.1	−21.9	
3000	6.0	−5.7	−22.7	
**Shrub density**				
100–3000	−19.0	−24.6	−24.4	

The 1977 survey was used as the base case for all comparisons. Positive values show an increase with Sample Period, while negative values show a decline. Note that because *Xmid* and *Scale* coefficients for shrub density did not vary with Sample Period, percent change estimates do not vary with distance from water.

### Ecological Functioning

We detected a strong relationship between ecological functioning (expressed as the ratio between areas of run-on and run-off) and both shrub volume (*R^2^* = 0.97; *F*_1,5_ = 157.30; *p*<0.001; Functionality = 0.83*e ^0.32 * Shrub volume^) and shrub density (*R^2^* = 0.82; *F*_1,5_ = 23.52; *p* = 0.005; Functionality = 0.37*e ^3.56 * Shrub density^). Hence, functionality increased rapidly with distance from water (Table 1; Fig. S1A) and reached an asymptote at 4890 m. Note, however, that this estimate extends beyond the sample transect and should be interpreted with caution. This process was correlated with an increase in the integrity of the organic-rich mounds that occur beneath patches of canopy shrubs (*R^2^* = 0.74; *F*_1,6_ = 13.93; *p* = 0.014; Functionality = 0.17+ (3.22 *Mound structure)). Only 2.5% of mounds near water were considered to be intact (i.e. patch area ≥ mound area; Table 1, Fig. S1B), and these were nearly 250% smaller than mounds recorded at 3000 m from water; 90.6% of mounds at the outer limit of sampling were intact.

## Discussion

AENP has a long history (nearly 40 years) of demonstrating elephant effects on ecosystem patterns and processes, and currently provides the most comprehensive account of these effects in South Africa (reviewed in [31]). Nevertheless, despite the contribution these accounts have made toward the larger debate on managing elephant impacts [20], the fact that impacts intensify in the vicinity of water (e.g. [2], [4], [21]), and the apparent vulnerability of succulent thicket to elephant [31], our study is the first to investigate these effects in relation to water in thicket. Furthermore, we provide the first explicit model of long-term variations in an elephant piosphere effect in a fenced system that may be used as a tool to monitor and manage the impact.

At our study site, we observed a clear spatial pattern in elephant effects, i.e. shrub structure increased rapidly to an asymptote with distance from water, which is consistent with other piosphere patterns (e.g. [14], [15], [22], [25], [26]). These results expand on the conclusions of Stuart-Hill [32] and Kerley, Tongway and Ludwig [28] who argued that the top-down foraging of elephant maintains the structure and ecological functioning of succulent thicket. We show that in the vicinity of water, and consequently with intensive utilization, the thicket shrub community is vulnerable to transformation as shrub patches are opened up, canopy volume declines and species that are less tolerant of elephant effects (e.g. those that recruit or regenerate poorly or are vulnerable to pollarding or uprooting - [47]) are gradually removed. This has significant implications for ecological functioning as the organic-rich mounds that occur beneath patches of shrubs are increasingly exposed and trapped resources run-off. The end-point is a highly transformed landscape adjacent to water, covered with a simple layer of ephemeral grasses and few of the structural elements that capture and utilize resources [28], [48]. Although our results are confounded by observations at a single water point (arrayed along a single axis), they are consistent with the patterns of transformation at other water points in AMC (determined using Normalized Difference Vegetation Indices (NDVIs) – [26]), and elsewhere in succulent thicket following intensive utilization by domestic browsers (e.g. [29], [32], [34], [35], [48]). Importantly, the latter studies show that a disturbance of the ecological processes in thicket, combined with generally slow regeneration dynamics, causes this trajectory of transformation to be virtually irreversible without active restoration. Thus, thicket landscapes with abundant water supply and elevated elephant numbers may be vulnerable to degradation (i.e. where ephemeral grasses dominate over woody shrubs, causing a decline in productivity and biodiversity – [29], [31], [34], [35]) as these patterns expand over time [7], [9]. Evidence from Hapoor water point support these predictions and show that shrub volume in particular has declined steadily at both the upper and lower limit of the piosphere pattern over the 31 year period of the survey; most striking is the roughly 300 m radial expansion of the grass-dominated habitats adjacent to water. Although Smith’s [26] analysis (using NDVIs on Landsat TM imagery) was limited to areas within 1 km from water in AMC (which excluded the estimation of asymptotes), she confirmed the expansion of these grass-dominated habitats at other water points. Not surprisingly, the extent and rate of expansion varied between points, mostly in accordance with their management history, but probably also in relation to other landscape features and barriers [4], [30]. The latter implies that the observed patterns may also not be symmetric at each point, causing inconsistencies in the shape of the piosphere pattern. For water points with management histories comparable to that of Hapoor (Fig. 1), grass cover reached distances of roughly 450 m from water, expanding by ∼300 m over a 16 year period. Lechmere-Oertel, Cowling and Kerley [48] argued that once the thicket system passes a threshold of self-restoration it loses resilience, thus tending toward an alternative state with reduced productivity [35]. We predict that elephant have the ability to expand the grassland-state across the landscape, and that this pattern of transformation can be interpreted using a state-and-transition model (*cf.* [49]). This suggests that attempts to use water availability as a tool to manage landscape heterogeneity in the presence of elephant (e.g. [5], [9], [12]) may be risky in succulent thicket that is vulnerable to such disturbances.

Elephant modify ecological patterns and processes at a range of scales [20], and while the patterns are often clear, the mechanisms may not be [23]. Although elephant piosphere effects are most apparent in the structure of woody communities, the scale of effects on associated biodiversity may be different from those observed for woody vegetation. Using modeled estimates from our final survey, we show that despite the clear relationship between shrub structure and ecological functioning in succulent thicket, the extent of elephant impacts at Hapoor water point varied between these features (displacement of asymptotes during 2008: shrub volume – ∼1070 m; shrub density – ∼3760 m; functionality – ∼4890 m, but see cautionary note); we presume that this reflects differences in the sensitivity of these features to elephant. Given that water and elephants are unevenly distributed across the landscape (e.g. [2]), it is likely that our estimates will vary between water points. Thus, it will be critically important to develop a predictive understanding of the relationship between the structural and functional attributes (or pattern and process) of ecosystems with elephant, which by definition are key aspects of ecological heterogeneity (*cf*. [50]). Failing this, attempts to use water availability as a tool to maintain landscape heterogeneity in the presence of elephant may fail in its objectives.

Although piosphere patterns generally expand with increased herbivore numbers and/or decreased rainfall, systems with multiple water points may show overlapping impacts, which reduce the extent of impact at each point (e.g. [7], [14], [15], [17]). During our 31-year study, we not only observed a decline in the thicket-dominated habitats adjacent to Hapoor, but also a significant decline in the displacement of asymptotes. Given that herbivory is the primary driver of thicket structure (as opposed to rainfall or fire, [34], [35], [51]) and that the elephant population [31] and water provisioning increased exponentially over the experimental period, we speculate that this decline reflects the overlap of impacts from neighboring water points. Similarly, we presume that in the absence of a change in rainfall, the closing of an adjacent water point during the late-1970s (M. Landman Unpublished data) would have released utilization pressure at this time and increased shrub volume (from ∼35% at 100 m to ∼6% at 3000 m) during the 1981-survey. Importantly, the patterns of overlap varied for shrub volume and density, with the former showing a steady decline (but see above) over the experimental period (thus continued transformation), and the latter a stabilization post-1981. The stabilization in shrub density reflects the fact that the rootstocks of some species remain intact (thus, also maintaining the shape of the sigmoid curve) with intensive utilization, which suggests that these species might recover following a release in utilization pressure (e.g. [34]). The consequences of this for ecological functioning and ecosystem resilience are not clear. Although we had no information on elephant numbers at Hapoor water point for the study, it is likely that our piosphere effects co-varied with these changes; furthermore, these impacts will co-vary with rainfall and other confounding variables (e.g. fire) in more dynamic systems, using more dynamic ecological features. Thus, it will be critically important to include these variables and their interactions in models that describe piosphere patterns in order to develop a predictive understanding of the mechanisms that create and maintain these patterns. We further show that elephant piosphere effects vary both spatially and temporally between ecological features (i.e. community composition, shrub volumes and densities, shrub species). This suggests that a more integrated understanding of the effects of elephant on ecological heterogeneity may be required before water availability is used to manage elephant effects.

Piosphere effects are usually considered model systems that provide key insights into the effects of herbivores on ecosystems (e.g. [7], [18]). Using multiple measures of biodiversity, we show that these effects are complex and that our ability to predict and manage such effects in the presence of elephant will be limited in the absence of long-term data. Instead we recommend an integrated multi-scaled approach to monitoring elephant effects in relation to water that incorporates both spatial and temporal variations and the structural and functional attributes of ecosystems. Furthermore, our findings clearly show the potentially adverse consequences of excessive water provisioning for succulent thicket communities [7]–[11]. This suggests that the current exceptionally dense network of water points in AMC (i.e. 12 water points within 120 km^−2^) likely compromises both biodiversity and conservation objectives [20], [31]. Elsewhere (e.g. Kruger National Park), negative relationships between abundant water supply, biodiversity and ecological resilience (e.g. [7], [10], [11], [13], [16]) have resulted in a review of water provisioning policies, and the subsequent closing of water points [5], [9], [12]. Our results caution against the establishment of additional water points in recently included novel habitats, and we advocate a significant reduction in water provisioning in AMC, albeit with greater impacts at existing water points.

## Supporting Information

Figure S1 Best-fit mixed-effects logistic growth models of (A) the ratio between areas of run-on and run-off, and (B) the proportion of intact mounds as a function of distance from water.
(TIF)
