# Supplementary figures and images for: Understanding Long-Term Variations in an Elephant Piosphere Effect to Manage Impacts
Source: PLoS One. 2012 Sep 17;7(9):e45334. doi: 10.1371/journal.pone.0045334 (PMC3444464; doi:10.1371/journal.pone.0045334)

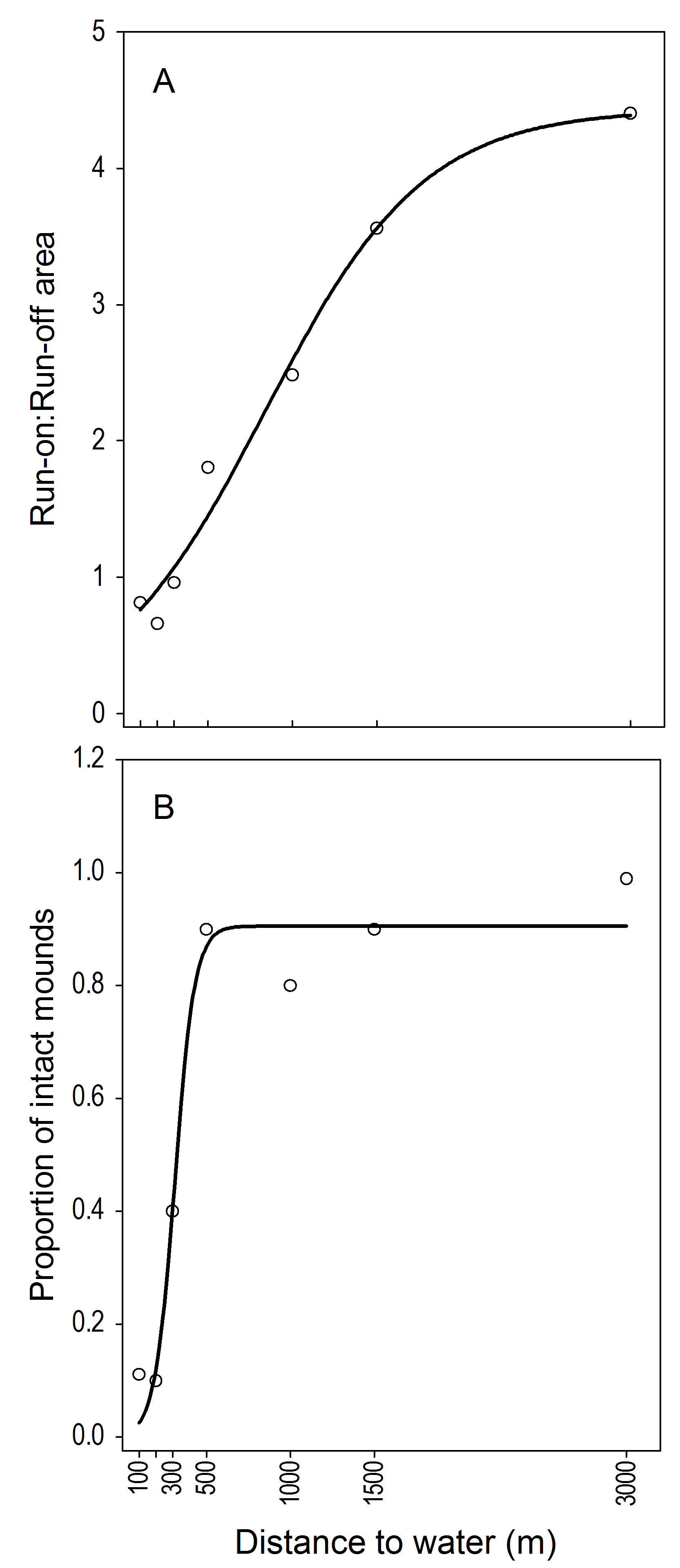

Supplement: Figure S1 — Best-fit mixed-effects logistic growth models of (A) the ratio between areas of run-on and run-off, and (B) the proportion of intact mounds as a function of distance from water. (TIF) [file pone.0045334.s001.tif]
